# Chronic In Vivo Biostability and Biocompatibility Evaluation of Polyether-Urethane-Based Balloon Implants for Cardiac Application in a Porcine Model

**DOI:** 10.3390/bioengineering13020168

**Published:** 2026-01-29

**Authors:** Min-Gi Kim, Jae-Young Seo, June-hong Kim, Jin-Chang Kim, Jun-Yong Park, Hyun-A Song, Kyeong-Deok Song, Min-Ku Chon

**Affiliations:** 1 Department of Polymer Science and Engineering, Pusan National University, Busan 46241, Republic of Korea; alsrl3529@pusan.ac.kr; 2 Department of Internal Medicine, School of Medicine, Pusan National University, Busan 49241, Republic of Korea; shope2024@pusan.ac.kr; 3 Department of Internal Medicine and Cardiology, Cardiovascular Center, Research Institute for Convergence of Biomedical Science and Technology, Pusan National University Yangsan Hospital, Pusan National University School of Medicine, Yangsan 50612, Republic of Korea; junehongk@gmail.com; 4 Department of Clinical Bio-Convergence, Pusan National University, Busan 46241, Republic of Korea; 5 Department of R&D Center, Tau Medical Inc., Busan 50612, Republic of Korea

**Keywords:** polyurethane, Pellethane, biocompatibility, biostability

## Abstract

Polyurethane-based implantable devices (PUIDs) delivered via catheter are increasingly used in structural heart interventions; however, limited in vivo data exist regarding their long-term biostability and biological safety. This study evaluated a balloon-shaped implant made of Pellethane^®^, a polyether-based polyurethane, designed as a three-dimensional intracardiac spacer and deployed via percutaneous femoral vein access. The device was chronically positioned adjacent to the tricuspid valve annulus in seven pigs for 24 weeks. Explanted devices and surrounding tissues were evaluated through material characterization (SEM, GPC, FT-IR, and ^1^H-NMR) and histological analysis. SEM and FT-IR confirmed preserved surface morphology and chemical bonds, GPC showed stable molecular weight, and ^1^H-NMR revealed intact urethane and ether linkages. Materials characterization revealed no evidence of hydrolytic or oxidative degradation, indicating structural stability of the devices. Histological analysis showed stable device positioning with minimal thrombosis or inflammatory response. Biocompatibility was confirmed via ISO 10993-1:2018 Standard (International Organization for Standardization (ISO): Geneva, Switzerland, 2018), and extractable substances were evaluated under exhaustive extraction conditions specified by ISO 10993-18:2020 (International Organization for Standardization (ISO): Geneva, Switzerland, 2020), with no toxicologically significant findings. These findings support the long-term biostability and biological safety of the PUIDs in dynamic cardiac environments, informing future design criteria for catheter-delivered cardiovascular devices.

## 1. Introduction

Polyether-based urethanes (PEUs) are thermoplastic elastomers widely used in implantable medical devices due to their favorable mechanical properties, biocompatibility [1,2,3], and ease of processing. Among these, Pellethane^®^, a medical-grade polyurethane [4], has been extensively applied in cardiovascular applications such as pacemaker leads [5,6], vascular grafts, catheters, and delivery sheaths. Its well-established hydrolytic stability under physiological conditions makes it suitable for long-term use in intravascular and subcutaneous environments, where long-term chemical and mechanical stability is critical [7,8].

Despite its clinical history, limited data exist on the long-term in vivo performance of PEUs under dynamic, blood-contacting environments like the heart. Although PEUs are resistant to hydrolysis [9,10], they are susceptible to oxidative degradation, particularly via reactive oxygen species generated during chronic inflammation. Hydrolysis typically leads to bulk degradation, whereas oxidation affects the surface, potentially compromising structural integrity. These degradation modes are influenced by local physiological conditions and the material’s morphology, making the implantation site and duration key factors in predicting material stability [11,12,13,14].

Polyether urethanes (PEUs) have been widely investigated for cardiovascular applications due to their unique combination of long-term hydrolytic stability and mechanical flexibility. Unlike bioabsorbable polymers, which undergo degradation through hydrolysis and may exhibit time-dependent loss of mechanical integrity, PEUs are designed to maintain structural stability during prolonged implantation. In contrast to metallic implants, which possess high stiffness and limited compliance, PEUs offer elastomeric behavior that better accommodates dynamic cardiac and vascular motion. These material characteristics make PEUs particularly suitable for blood-contacting intracardiac environments where sustained mechanical performance and biostability are required [15,16,17].

Previous studies have explored the chemical stability and degradation behavior of PEUs using in vitro accelerated aging models [18,19]. However, there remains a lack of comprehensive data regarding the long-term chemical and morphological integrity of these materials following implantation in dynamic biological environments such as the heart. In particular, hydrolytic and oxidative stress factors in vivo need to be evaluated separately from previous devices [9], as they may be affected differently by the surrounding environment, such as blood flow, pulsatile shear stress, and inflammatory responses, as well as by the type of device manufactured [20,21].

In this study, a polyether-based urethane implantable device (PUID) composed of Pellethane^®^ was chronically implanted adjacent to the tricuspid valve annulus, maintaining continuous contact with the right atrial wall under physiological cardiac motion [22,23]. The study evaluated thrombosis, inflammation, and material degradation, along with histological analysis of adjacent cardiac tissues.

To further assess the material’s long-term biostability, the recovered Pellethane^®^ component 24 weeks after implantation was analyzed using scanning electron microscopy (SEM), Gel Permeation Chromatography (GPC), Fourier-Transform Infrared Spectroscopy (FT-IR), and nuclear magnetic resonance (^1^H-NMR) according to ISO 5910:2024 [24].

Additional supporting data were obtained through extended chemical characterization, ISO10993 biocompatibility [25,26,27,28], hemocompatibility, extraction, and leaching toxicological assessment [29,30,31].

This study comprehensively evaluated thrombosis, inflammatory response, material degradation, and histological changes in adjacent cardiac tissues following long-term implantation of a polyether-urethane-based intracardiac device.

The integrated evaluation combining biological assessment and material biostability aims to establish comprehensive performance data for Pellethane^®^-based devices in long-term, blood-contacting, catheter-delivered applications.

The tricuspid valve annulus was selected as the implantation site because the investigated device was designed as a three-dimensional intracardiac spacer intended to reside at the valve plane and interact with the regurgitant orifice region under dynamic cardiac motion. This study does not evaluate an FDA-approved therapeutic device, nor does it aim to demonstrate clinical efficacy. Rather, it represents a preclinical, material-focused investigation of the chronic in vivo biostability and biocompatibility of a polyether-urethane-based intracardiac implant concept. The implantation site was selected to reflect anatomical and mechanical conditions relevant to contemporary transcatheter therapies for tricuspid regurgitation, while FDA-approved devices are referenced solely to provide clinical context rather than direct comparison.

## 2. Materials and Methods

### 2.1. Materials

**Figure 1:**
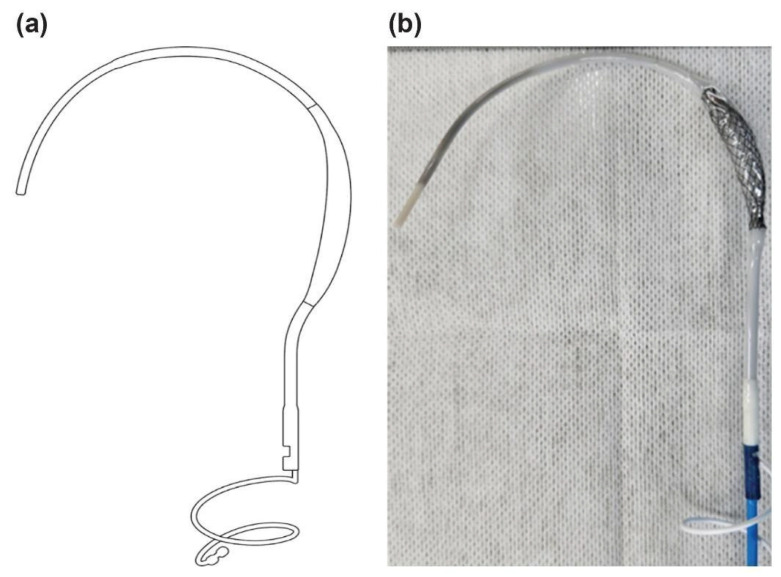
External structure of the PUID. (**a**) Schematic illustration; (**b**) Photograph of the actual device.

The balloon spacer and shaft components of the **PUID** were made from Pellethane^®^ (Emerald extrasion service, Chicago) and thermoformed following a controlled fabrication process to ensure dimensional consistency. Although the PUID includes other components, the analysis in this study focused specifically on the Pellethane^®^-based elements. Control samples were prepared using the same fabrication process, and all specimens were EO-sterilized prior to testing (Figure 1).

### 2.2. Methods

#### 2.2.1. Animal Study Design

Seven conventional farm pigs (domestic pigs; mean age 14 weeks, mean weight 38.8 ± 0.8 kg), consisting of three females and four males, underwent femoral vein catheterization for PUID implantation at the tricuspid annulus under fluoroscopic guidance (Integris H5000F; Philips Medical Systems, Amsterdam, The Netherlands). The porcine model was selected because porcine cardiac anatomy, myocardial tissue characteristics, and healing responses are well recognized to closely resemble those of humans, making it a relevant preclinical model for evaluating local histological responses and tissue compatibility of intracardiac implantable devices. PUIDs positioning was continuously monitored using fluoroscopy and echocardiography [22]. During the 24-week survival period, animals received daily oral rivaroxaban (20 mg) to reduce thrombotic risk. At the end of the study, all animals were euthanized, and their hearts and surrounding tissues were explanted for comprehensive histopathological and material analysis. All procedures were conducted in accordance with NIH guidelines and the Animal Care and Use Committee policies of Pusan National Yangsan University Hospital (PNUYH), with study protocols approved by the PNUYH IRB (No. 2021-007-A1C0[0]).

#### 2.2.2. Histological Analysis

Gross necropsy was performed at the time of explantation to assess device positioning and surrounding cardiac tissues. Immediately after necropsy, all explanted tissues were fixed in formalin without delay and subsequently processed for histological analysis to preserve tissue morphology and inflammatory features. Cardiac tissues were then paraffin-embedded and sectioned at 5 µm thickness for hematoxylin and eosin (H&E) staining. Histopathological evaluation focused on the presence of fibrosis, histiocytic infiltration, hemorrhage, and inflammatory responses.

#### 2.2.3. Analytical Methods

To evaluate the in vivo chemical and morphological stability of the balloon spacer component of the PUID, which was fabricated from Pellethane^®^ 80A, samples were subjected to a series of standardized analytical techniques. Devices retrieved after 24 weeks of implantation were rinsed with physiological saline, air-dried for 48 h, and the balloon spacer component was sectioned into approximately 1 cm × 1 cm pieces (T1: implanted samples for 24 weeks). For comparison, non-implanted control specimens of the same PEU balloon spacer, fabricated using an identical manufacturing process, were prepared (T0: non-implanted controls). All analytical evaluations, including surface imaging, chemical structure analysis, and molecular weight determination, were conducted using both T0 and T1 samples.

Scanning Electron Microscopy (SEM) was performed using a Sigma 300VP (Carl Zeiss Microscopy GmbH, Oberkochen, Germany) after platinum (Pt) coating to evaluate surface morphology and identify signs of microcracking, delamination, or general surface degradation. Imaging was conducted at magnifications of ×500 and ×1000 across three predefined regions of each sample: immediate, proximal, and distal sections.

Fourier-Transform Infrared Spectroscopy (FT-IR) was performed in attenuated total reflectance (ATR) mode using a Nicolet iS50 spectrometer (Thermo Fisher Scientific, Waltham, MA, USA) to assess the chemical structural integrity. Absorbance spectra were collected in the range of 600–4000 cm^−1^ with a resolution of 4 cm^−1^. Comparative analysis between implanted and non-implanted samples was conducted to identify potential oxidative or hydrolytic modifications, with particular attention to characteristic peaks corresponding to urethane, ether, and alkyl groups.

Gel Permeation Chromatography (GPC) was employed to evaluate changes in molecular weight distribution. The analysis was performed using a Waters ACQUITY APC System with dimethylformamide (DMF) as the eluent at a flow rate of 0.3 mL/min. The column temperature was maintained at 40 °C, and detection was carried out using a refractive index (RI) detector. The injection volume was 50 μL, with a total run time of 30 min. Calibration was conducted using polystyrene standards. The number-average molecular weight (M_n_) and weight-average molecular weight (M_w_) were calculated to assess potential polymer chain scission or crosslinking induced by in vivo exposure.

^1^H-NMR spectroscopy was performed using a Bruker Avance Neo 600 MHz solid-state ^1^H-NMR spectrometer (Bruker Corporation, Billerica, MA, USA) with deuterated tetrahydrofuran (THF-d_8_) as the solvent. Spectra were acquired for both implanted and control samples to evaluate urethane bond stability, detect any new chemical shifts, and assess the overall integrity of the molecular structure.

All procedures were conducted under consistent conditions using both qualitative and quantitative chemical characterization methods, as outlined in ISO 10993-18:2020. In addition, literature-based information was incorporated, where appropriate, to complement the experimental data in accordance with ISO 10993-18:2020.

#### 2.2.4. Biocompatibility Testing Method

Biocompatibility testing was conducted using a fully sterilized device featuring a balloon structure composed of Pellethane^®^. The PUID was manufactured using the company’s proprietary production process to accurately represent actual patient exposure. All biocompatibility testing was conducted in accordance with ISO 10993-1:2018 and Good Laboratory Practice (GLP) standards. The studies were performed by GLP-certified laboratories NAMSA (North American Science Associates, LLC) and Nelson Laboratories ensuring both data reliability and compliance with relevant regulatory requirements.

A comprehensive suite of biocompatibility evaluations was carried out, and the results are summarized in Table 1.

#### 2.2.5. Chemical Characterization

Chemical characterization was conducted by Eurofins Munich, in accordance with ISO 10993-17:2023 and ISO 10993-18:2020, to assess potential toxicological risks. In accordance with ISO 10993-18:2020, the chemical characterization was performed using exaggerated and exhaustive extraction conditions rather than simulated-use extraction.

Following calculation of the device surface area, a surface area-to-extraction volume ratio of 6 cm^2^/mL was applied, consistent with the recommendations of ISO 10993-12 and ISO 10993-18. This ratio was conservatively selected based on the intended clinical use of the device, as well as the presence of components with varying thicknesses and geometries that may influence extraction efficiency.

To simulate clinically relevant conditions, consecutive extractions were carried out in water (polar), isopropanol (semi-polar), and n-hexane (nonpolar) at 50 °C for 24 h per extraction. The test materials were agitated during extraction to enhance release. A total of five consecutive extractions were performed for each solvent until nonvolatile residues were no longer detected, thereby representing a worst-case exposure scenario for toxicological risk assessment.

The resulting extracts were then analyzed using the techniques recommended in ISO 10993-18:2020 (Chemical characterization of medical device materials within a risk management process), specifically those listed below. Test methods for establishing the material composition of medical device materials of the standard.

Gas Chromatography–Mass Spectrometry (GC-MS): GC-MS was used to analyze semi-volatile organic compounds. Semi-quantitative analysis was performed by external calibration using surrogate standards with retention times closest to the detected analytes.

Inductively Coupled Plasma Mass Spectrometry (ICP-MS): ICP-MS was applied to the final extracts in phosphate-buffered saline (PBS) to quantify elemental and inorganic constituents.

Liquid Chromatography–Mass Spectrometry (LC-MS): Employed to detect polar and thermally labile compounds not amenable to GC-MS analysis. LC-MS results complemented the GC-MS data by identifying additional low-volatility substances.

Substance identification was supported by spectral matching using both the NIST mass spectral library and the proprietary internal database of Eurofins Munich. The highest intensity peaks observed in each solvent system were prioritized for identification.

Threshold-based evaluations were conducted using AET, PDE, and TTC approaches in accordance with ISO 10993-18:2020 and ISO 10993-17:2023, and Margin of Safety (MoS) values were calculated for substances with established exposure limits. The derivation of PDE values followed toxicological risk assessment principles defined in ISO 10993-17:2023, while TTC was applied based on internationally accepted threshold-of-toxicological-concern concepts referenced within the same standard.

This multi-technique analytical approach ensured the comprehensive profiling of extractable chemical constituents in compliance with international standards, supporting the risk assessment of the material’s suitability for prolonged or permanent contact with human tissues and body fluids.

#### 2.2.6. Statistical Analysis

This study was designed as a pilot preclinical investigation; therefore, no formal a priori sample size calculation or power analysis was performed. The study focused on descriptive assessment of long-term in vivo biostability and biocompatibility rather than hypothesis-driven efficacy testing.

All quantitative data obtained from animal experiments and material characterization were summarized using descriptive statistics, including mean ± standard deviation. Given the exploratory nature of the porcine study and the limited sample size (n = 6), no formal hypothesis testing or inferential statistical comparisons were performed. Instead, reproducibility and consistency across animals were assessed by evaluating variability trends for device positioning, histological scoring, and analytical measurements (SEM, FT-IR, GPC, and ^1^H-NMR). All statistical analyses and data tabulations were conducted using standard spreadsheet-based tools, and results were interpreted qualitatively to determine whether implanted samples deviated meaningfully from non-implanted controls. This statistical approach is consistent with preclinical large-animal studies focused on biostability and material integrity rather than hypothesis-driven comparative testing.

## 3. Results

### 3.1. Device Stability and Positioning

All seven farm pigs underwent successful implantation of PUIDs via a percutaneous femoral vein approach. Correct positioning of the devices at the tricuspid annulus within the right atrium was verified by both fluoroscopy and echocardiography. Over the 24-week observation period, the implants remained stable without evidence of migration, displacement, rupture, or mechanical failure.

One animal was excluded from the final analysis due to an infection identified at 3 weeks post-implantation. During follow-up, the animal exhibited systemic deterioration, and blood culture analysis detected *Streptococcus canis*, a non-specific β-hemolytic streptococcal species commonly associated with dogs and cats and known to originate from external environmental exposure. Based on veterinary assessment, the infection was considered unlikely to be device-related. To avoid confounding effects of infection-related systemic inflammation on the evaluation of thrombosis, inflammatory response, and local histological outcomes, this animal was excluded from further analysis. The remaining six animals maintained consistent device integrity and positioning throughout the study.

### 3.2. Histological Findings

**Figure 2:**
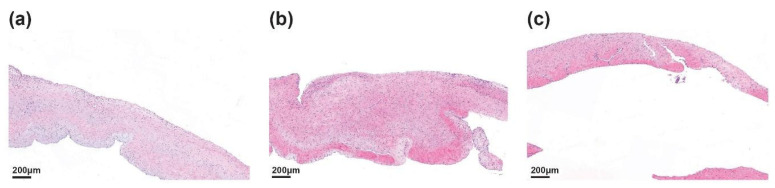
Pathological findings of the tricuspid valve in a porcine heart 24 weeks after implantation of the PUID. (**a**) Anterior tricuspid leaflet showing preserved architecture with moderate focal thickening. (**b**) Posterior tricuspid leaflet demonstrating minimal localized tissue response near the implant interface. (**c**) Septal tricuspid leaflet exhibiting intact structure without evidence of chronic inflammation or adverse remodeling.

Histological examination of explanted heart tissues at the 24-week endpoint revealed favorable biocompatibility of the PUID. Baseline (t = 0) histological analysis was not performed, as no tissue excision or device implantation occurred at baseline and the study was designed to evaluate chronic tissue responses following long-term intracardiac implantation. Interpretation of post-implant findings was therefore performed relative to well-established normal porcine cardiac histology reported in the literature Figure 2.

Histopathological evaluation was conducted on explanted porcine hearts 24 weeks post-implantation of PUIDs. Histological evaluation of the anterior tricuspid leaflet (Figure 2a) demonstrated preserved overall architecture with moderate focal thickening and limited histiocytic activity. The posterior leaflet (Figure 2b) generally maintained structural integrity with only minor focal tissue responses near the implant interface. The septal leaflet (Figure 2c) appeared structurally intact without evidence of chronic inflammation or adverse remodeling. Inflammatory or foreign body reactions were limited and confined to specific sites, indicating stable tissue compatibility over time. The septal leaflet appeared structurally intact without signs or architectural distortion. No notable chronic inflammation or adverse remodeling was observed in this region, suggesting stable interaction with the surrounding cardiac tissue.

### 3.3. Biocompatibility

The medical device, the PUID manufactured in accordance with internal process protocols, was subjected to extraction testing based on surface area to reflect patient exposure. A biological safety assessment was conducted in compliance with ISO 10993 standards and Good Laboratory Practice (GLP) regulations. A summary of each test is presented in Table 1 below.

Biological safety and chemical characterization assessments are essential to confirm patient safety prior to clinical evaluation and to ensure the in vivo biocompatibility of implantable medical devices.

### 3.4. Toxicological Assessment

In this study, the release of nonvolatile residues (NVRs) and semi-volatile organic extractables from the test article specifically, the PUID was analyzed following liquid extraction.

The highest detected peaks in each of the three solvents were characterized using the NIST mass spectral library and Eurofins Munich’s internal material library. Chemical analysis of the test article extracts identified semi-volatile, nonvolatile, and elemental substances that may be released under laboratory conditions. Most of the detected compounds were found at levels below the analytical evaluation threshold (AET) or the permitted daily exposure (PDE) limits and therefore did not require further toxicological assessment.

Due to the consistent patterns observed in the chromatographic profiles, the 50 most prominent peaks in each extractables study were selected for characterization. These peaks were shown to represent structurally related compounds, primarily consisting of monomeric or oligomeric derivatives. Among these, four distinct compounds were detected at concentrations exceeding the AET in extracts obtained using isopropanol and hexane, excluding water. Therefore, a comprehensive toxicological risk assessment was conducted in accordance with ISO 10993-17:2023.

Each of the four substances was fully identified and associated with a CAS number, enabling toxicological reference checks in established databases such as PubChem, ECHA, GESTIS, and INCHEM. Following identification, toxicological limit values were retrieved from the literature, with preference given to studies closely aligned with the clinical use of the medical device particularly in terms of exposure route, duration, and species. Oral or parenteral thresholds were prioritized when available.

Subsequently, tolerable intake (TI) values were calculated for each substance using reference points such as NOAEL, LOAEL, DNEL, or TDI. NOAEL values were obtained from relevant references, and the corresponding numerical values and sources are provided in Table 2. A default uncertainty factor of 10 was applied to account for differences between the experimental conditions in the literature and the intended clinical application of the device. These TI values were then compared to the estimated worst-case exposure levels to derive the Margin of Safety (MoS). In all cases, the MoS values exceeded 1, indicating an acceptable safety margin.

The values derived for TI and EEDmax for the four substances are presented in the table below.

**Table 2:** Determination of the tolerable intake (TI) and the estimation exposure dose (EEDmax) and MoS (Margin of Safety).

Solvent (Analysis)	Substance	NOAEL [mg/kg BW/day] [32,33,34]	TI [µg/kg BW/day]	EEDmax [µg/kg Bw/day]	MoS [TI/EEDmax]
Isopropanol (GC-MS)	Butylated Hydroxytoluene	25	25	0.47	53.19
	Erucamide	1000	1000	0.02	50,000
	Octadecyl 3-(3,5-di-tert-butyl-4-hydroxyphenyl) propionate	64	64	0.97	65.97
n-Hexane (GC-MS)	Butylated Hydroxytoluene	25	25	0.20	125
	Erucamide	1000	1000	0.01	100,000
	Octadecyl 3-(3,5-di-tert-butyl-4-hydroxyphenyl) propionate	64	64	0.32	200
n-Hexane (HPLC-MS)	Irganox 1076 (Octadecyl 3-(3,5-di-tert-butyl-4-hydroxyphenyl) propionate)	64	64	43.46	1.47

Additionally, unidentified extractables primarily hydrolysis products of polymeric materials were not considered toxicologically relevant. These substances were further assessed through implantation and biocompatibility studies, all of which showed no signs of toxicity or mutagenicity. Taken together, both the four AET-exceeding substances and all other extractables were determined to pose no toxicological risk under the tested conditions.

### 3.5. Scanning Electron Microscopy (SEM)

**Figure 3:**
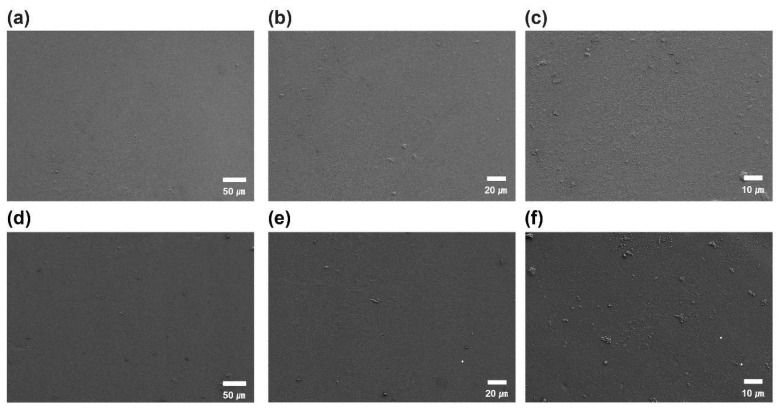
Comparison of scanning electron microscopy (SEM) images of the PEU balloon spacer surface between the T0 sample (non-implanted control) and the T1 sample (implanted for 24 weeks). SEM imaging was performed using a Sigma 300VP system (Carl Zeiss Microscopy Ltd., UK) at an accelerating voltage of 5 kV after platinum (Pt) coating. Images were acquired at magnifications of 500×, 1000×, and 2000×, with corresponding scale bars indicated in each image. The midsection of the balloon spacer was examined to evaluate surface morphology and potential degradation features. (**a**) T0, 500×; (**b**) T0, 1000×; (**c**) T0, 2000×; (**d**) T1, 500×; (**e**) T1, 1000×; (**f**) T1, 2000×.

High-resolution images of the material surface were used to assess the presence of brittleness and surface cracking. SEM analysis showed that the surface morphology of T1 samples was well maintained after 24 weeks of implantation. The surface remained largely smooth and intact, with no evidence of cracking, brittleness, or severe erosion, closely resembling that of the T0 samples. No signs of oxidative or hydrolytic degradation were observed in the T1 samples during the implantation period (Figure 3).

Minor surface rough features observed after implantation are considered expected for polyurethane materials exposed to long-term blood-contacting and mechanically dynamic intracardiac conditions. These features are likely associated with protein adsorption, minor surface abrasion from cyclical cardiac motion, and superficial biological deposition, rather than structural damage or bulk material degradation. This interpretation is supported by the absence of microcracking or delamination on SEM and by complementary chemical and molecular weight analyses.

### 3.6. Fourier-Transform Infrared Spectroscopy (FT-IR)

Fourier-Transform Infrared Spectroscopy (FT-IR) of the implanted PEU grafts demonstrated that the chemical structure of the material was preserved after 24 weeks of intracardiac implantation. The C=O stretching vibration around 1700 cm^−1^ and the combined N–H bending and C–N stretching near 1530 cm^−1^ were well maintained in T1 samples, showing strong similarity to T0 samples, indicating that the core urethane structure remained intact after implantation. In addition, peaks in the 1100–1300 cm^−1^ region, corresponding to ether (C–O–C) and C–N vibrations, showed minimal variation, supporting the stability of the polymer backbone.

**Figure 4:**
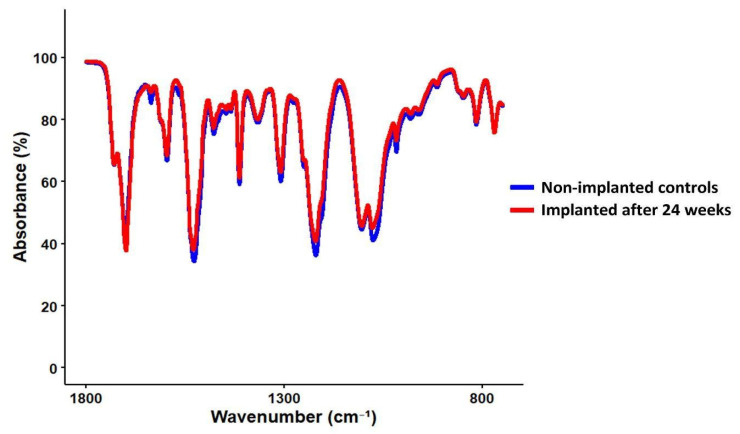
Comparison of FT-IR spectra between T1 samples (PEU balloon spacers implanted for 24 weeks) and T0 samples (non-implanted controls).

The characteristic absorption bands associated with urethane (-NH, -C=O), ether (C-O-C), and alkyl (C-H) groups remained stable, with no significant peak shifts or formation of new bands. Moreover, no additional changes were observed in T1 samples compared to the spectra obtained from T0 samples (Figure 4).

### 3.7. Gel Permeation Chromatography (GPC)

**Figure 5:**
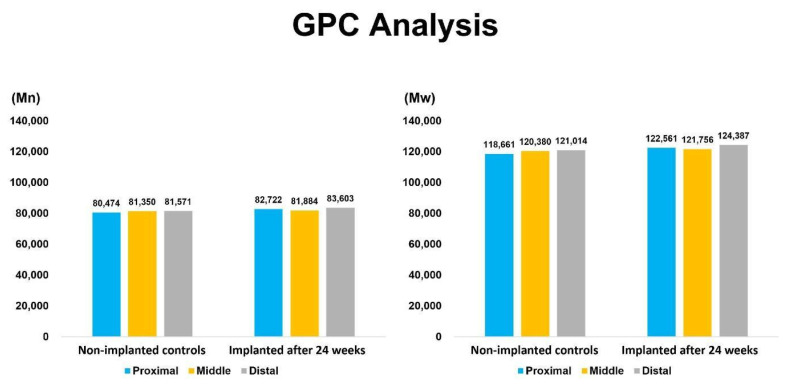
Comparison of GPC chromatograms of T1 samples (PEU balloon spacers implanted for 24 weeks) and T0 samples (non-implanted controls).

Gel Permeation Chromatography (GPC) analysis demonstrated that the number-average molecular weight (Mn) and weight-average molecular weight (Mw) of T1 samples after 24 weeks of implantation were comparable to those of T0 samples. The GPC analysis was conducted as a qualitative and comparative assessment of molecular weight distribution between T0 and T1 samples, and was not intended as a statistical evaluation based on repeated measurements; therefore, error bars are not applicable (Figure 5).

### 3.8. Nuclear Magnetic Resonance (^1^H-NMR)

Nuclear magnetic resonance (^1^H-NMR) spectroscopy of T1 samples after 24 weeks of implantation exhibited well-resolved signals assignable to distinct proton environments within the polymer. Aromatic protons appeared at 7.2–7.6 ppm, with para- and meta-substituted protons assigned to 7.2–7.4 ppm and 7.4–7.6 ppm, respectively. Methylene protons adjacent to the urethane linkage (–CH_2_–NH–COO–) were observed at approximately 4.0 ppm. Signals in the range of 3.4–3.8 ppm were attributed to ether-linked methylene groups or methylene units within the poly tetramethylene oxide (PTMO) backbone. The urethane N–H proton was observed at 8.1–8.4 ppm.

**Figure 6:**
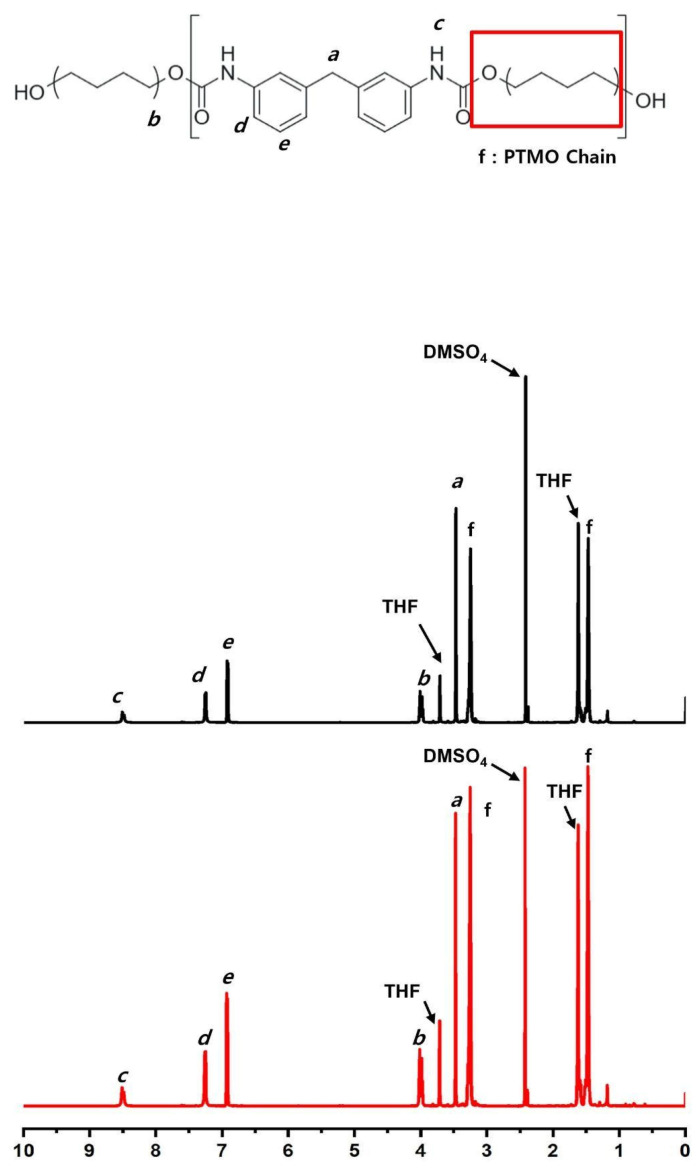
^1^H-NMR comparison between T1 samples (PEU balloon spacers implanted for 24 weeks) and T0 samples (non-implanted controls).

These characteristic signals showed no significant chemical shift changes or appearance of new peaks. The spectral profiles of T1 samples were comparable to those of T0 samples, with no additional signals indicating chemical alteration (Figure 6).

## 4. Discussion

This study was designed as a material-focused preclinical investigation to evaluate the long-term in vivo biostability of Pellethane^®^ used in the balloon spacer component and the overall biocompatibility of the fully assembled PUID device, with the aim of generating foundational safety data to support future device development rather than assessing clinical performance or regulatory equivalence.

The present study integrates assessment of thrombosis, inflammation, material degradation, and histological responses of adjacent cardiac tissues to provide a comprehensive evaluation of in vivo biostability and biocompatibility.

Informed by prior studies on the original Pivot Mandu device, which featured a three-layered structure composed of an inner polyurethane balloon, a nitinol mesh support, and an outer ePTFE coating, we implemented a key design modification in this study: the removal of the outer ePTFE layer. This change allowed us to evaluate the long-term durability and chemical stability [5,7,8] of Pellethane^®^ as the sole exposed balloon material, thereby simplifying the device structure and focusing on the critical performance of the polyurethane membrane under physiological conditions. Moreover, the removal of the ePTFE layer resolved the elastic mismatch issue that could otherwise create interlayer gaps, providing a justification for the design change and ensuring consistent conformity to the intended target size.

Through a comprehensive 24-week preclinical study in a porcine model, we combined histological evaluation with analytical techniques to evaluate the biological and physicochemical responses to the implanted device. The device remained stably positioned in all animals (excluding one excluded due to procedural infection), without evidence of structural failure, migration, or mechanical compromise. This is particularly important because intracardiac devices are continuously subjected to cyclical mechanical stress from cardiac motion. Fluoroscopic and echocardiographic assessments confirmed stable positioning throughout the implantation period, demonstrating mechanical compatibility with dynamic intracardiac anatomy.

Histological analysis showed only mild and localized tissue responses. H&E staining revealed limited fibrotic encapsulation, minimal histiocytic infiltration, and localized inflammation at the tissue implant interface. Notably, endothelialization was observed in several specimens, suggesting integration potential without inducing excessive inflammatory or foreign body responses. These results support the biological inertness of the single layer Pellethane^®^ balloon spacer in a high-shear blood-contacting environment.

Parallel to tissue-level evaluation, material degradation was assessed using a series of orthogonal analytical techniques. SEM analysis showed no evidence of microcracking, delamination, or surface embrittlement, indicating minimal surface-level oxidative degradation [5,35]. FT-IR spectra of explanted samples confirmed the preservation of characteristic urethane and ether peaks, with no appreciable loss in absorption intensity. These findings confirm that the polymer’s chemical structure remained intact after 24 weeks of implantation.

GPC analysis revealed stable number-average and weight-average molecular weights (Mn and Mw), indicating no detectable bulk degradation, and confirming the polymer’s resistance to hydrolysis [5,9] over the study period. ^1^H-NMR analysis showed no chemical shift changes or signal pattern alterations, reinforcing the absence of chain scission or chemical rearrangement.

These findings are consistent with previous reports on the biostability of Pellethane^®^. Polyether based urethanes (PEUs), such as Pellethane^®^, are well recognized for their strong resistance to hydrolytic degradation, a property supported by long-term clinical data showing no significant molar mass reduction even after more than a decade of implantation [5]. This hydrolytic stability is particularly critical for long-term implantable devices, as bulk degradation driven by hydrolysis can severely compromise mechanical performance [35]. Although oxidative degradation has been observed in clinically retrieved PEU-based devices, its effects are largely limited to the surface and do not significantly impact overall device function. The absence of substantial chemical or mechanical degradation over time supports the design decision to simplify the original Pivot Mandu configuration by removing the outer ePTFE layer.

In addition to material biostability evaluation, biocompatibility testing was performed in accordance with ISO 10993-1 and ISO 10993-18 standards. No cytotoxicity, genotoxicity, or systemic toxicity was observed, and all extractables remained within acceptable analytical evaluation threshold (AET) and permitted daily exposure (PDE) limits.

Collectively, this study provides strong evidence that the modified Pellethane^®^-based PUID maintains structural and chemical stability under chronic intracardiac conditions. By integrating histopathological, mechanical, and chemical analyses, we offer a comprehensive perspective on the in vivo behavior of polyurethane-based materials in long-term blood-contacting applications. This simplified design, eliminating surface coatings while preserving biocompatibility and durability, supports the future use of PEUs in catheter-delivered, permanent cardiac implants.

Further exploration is needed to integrate polyether urethanes (PEUs) with metallic components and bioabsorbable polymers, as most cardiovascular medical devices are inherently multicomponent systems. In such devices, metallic materials primarily provide structural support and load-bearing capability, whereas PEU layers contribute the flexibility and compliance required to accommodate pulsatile vascular and intracardiac motion. Bioabsorbable polymers may further enhance device performance by enabling temporary scaffolding, controlled degradation, or delivery-phase functionality during early healing stages. While the present study systematically addressed the long-term biostability and wear behavior of PEUs, fatigue-, corrosion-, and interface-related interactions between multiple materials must ultimately be evaluated in an integrated manner at the device level to support clinical translation [9,10,11,36].

## 5. Conclusions

The 24-week preclinical study of the PUID demonstrated excellent in vivo biostability and biocompatibility when used as an intracardiac spacer for transcatheter tricuspid valve intervention. The device maintained its molecular integrity, structural morphology, and surface characteristics, with no significant degradation or adverse host tissue response. These findings support the use of Pellethane^®^ as a durable material platform for future catheter-based cardiovascular devices.

## Figures and Tables

**Table 1:** Biocompatibility test summary.

Test Description	Relevant Std	Results	Test Institute
Cytotoxicity with Quantitative Evaluation (MTT)	ISO 10993-5:2009	No significant cytotoxicity observed; cell viability exceeded 70% compared to control, meeting ISO 10993-5 criteria. (PASS)	Nelson labs
Intracutaneous Reactivity test	ISO 10993-23:2021 (International Organization for Standardization (ISO): Geneva, Switzerland, 2021.)	No significant intracutaneous irritation observed in rabbits, meeting ISO 10993-10 and ISO 10993-23 criteria. (PASS)	Nelson labs
Hemolysis test	ISO 10993-4:2017 (International Organization for Standardization (ISO): Geneva, Switzerland, 2017) ASTM F756-17 (ASTM International: West Conshohocken, PA, USA, 2017.)	No significant hemolytic activity observed; hemolysis levels were well below 2%, meeting ASTM criteria. (PASS)	Nelson labs
Partial Thromboplastin time test	ISO 10993-4:2017 ASTM F2382-18 (ASTM International: West Conshohocken, PA, USA, 2018.)	No significant effect on plasma coagulation time observed, meeting ASTM F2382 requirements. (PASS)	NAMSA
Platelet and Leucocyte count test	ISO 10993-4:2017	No significant effect on platelet count observed, meeting ASTM and ISO hemocompatibility criteria. (PASS)	NAMSA
Complement Activation test	ISO 10993-4:2017	No significant complement activation observed compared to controls, meeting ISO 10993-4 requirements. (PASS)	Nelson labs
Sensitization test	ISO 10993-10:2021 (International Organization for Standardization (ISO): Geneva, Switzerland, 2021)	No sensitization reactions observed in guinea pigs, meeting ISO 10993-10 requirements. (PASS)	Nelson labs
Pyrogen test	ISO 10993-11:2017 (International Organization for Standardization (ISO): Geneva, Switzerland, 2017.) USP–NF-<151>(2017 United States Pharmacopeial Convention (USP): Rockville, MD, USA, 2017.)	No significant pyrogenic response observed, meeting ISO 10993-11 acceptance criteria. (PASS)	Nelson labs
Acute Systemic toxicity test	ISO 10993-11:2017 (International Organization for Standardization (ISO): Geneva, Switzerland, 2017.)	No acute systemic toxicity observed over 72 h; all animals survived with no significant clinical or necropsy findings. (PASS)	Nelson labs
Sub Chronic toxicity test	ISO 10993-11:2017	No subacute or sub-chronic systemic toxicity observed; findings related to administration, not test article. (PASS)	Nelson labs
Subcutaneous 13-week Implant test	ISO 10993-6:2016 (International Organization for Standardization (ISO): Geneva, Switzerland, 2016.)	Acceptable local tissue compatibility demonstrated; minimal inflammation, normal capsule formation, and no significant pathology observed. (PASS)	NAMSA
Bacterial Reverse Mutation test (AMES) test	ISO 10993-3:2014 (International Organization for Standardization (ISO): Geneva, Switzerland, 2014.)	No mutagenic activity observed in bacterial reverse mutation assay, with or without metabolic activation. (PASS)	Nelson labs
Chromosome Aberration test	ISO 10993-3:2014	No significant chromosomal aberrations observed in mammalian cells, indicating no clastogenicity. (PASS)	Nelson labs
Chronic Toxicity test	ISO 10993-11:2006	No evidence of chronic toxicity based on risk assessment and study data, supporting waiver of chronic toxicity testing per ISO 10993-1 and FDA guidance. (PASS)	Eurofins
Carcinogenicity test	ISO 10993-3:2003	No evidence of carcinogenicity based on genotoxicity results, chemical characterization, literature, and implantation data, in line with ISO 10993-1 and FDA guidance. (PASS)	Eurofins

Abbreviations: MTT, 3-(4,5-dimethylthiazol-2-yl)-2,5-diphenyltetrazolium bromide; ISO, International Organization for Standardization; ASTM, ASTM International; USP–NF, United States Pharmacopeia–National Formulary; AMES, bacterial reverse mutation assay.

## Data Availability

The original contributions presented in this study are included in the article. Further inquiries can be directed to the corresponding author.
